# A new species of *Nesolotis* Miyatake, 1966 (Coccinellidae, Coccinellinae, Sticholotidini) from Laos

**DOI:** 10.3897/BDJ.12.e129927

**Published:** 2024-07-15

**Authors:** Lanlan Lv, Xingmin Wang

**Affiliations:** 1 College of Plant Protection, South China Agricultural University, Guangzhou, China College of Plant Protection, South China Agricultural University Guangzhou China; 2 Engineering Research Center of Biological Control, Ministry of Education, Guangzhou, China Engineering Research Center of Biological Control, Ministry of Education Guangzhou China

**Keywords:** Coccinellidea, *Nesolotis*, new species, new distribution, Laos

## Abstract

**Background:**

*Nesolotis* Miyatake, 1966 shows a high diversity in the Oriental Realm.

**New information:**

Here, we recorded this genus in Laos for the first time and provided a detailed description of a new species, namely *Nesolotislaotica* Lv & Wang, **sp. nov.**

## Introduction

The genus *Nesolotis* was established by Miyatake (1966), based on specimens found in Japan, with the description of type species *Nesolotisimpunctata* Miyatake, 1966. Hoàng (1982) subsequently erected another genus *Paranesolotis* from Vietnam. These two genera (*Nesolotis* and *Paranesolotis*) were later proposed as synonyms of *Sticholotis* by Ślipiński (2004), based on their 10-segmented antennae. However, Wang et al. (2010) and Wang et al. (2017) re-validated *Nesolotis*, based on a comprehensive study of the species from China, indicating a series of morphological differences between *Nesolotis* and *Sticholotis*. Currently, 17 species have been recorded worldwide, strictly occurring in the Oriental and Palaearctic Realms. However, it exhibits high diversity in the Oriental Realm, with 16 species recorded in this region. (Kamiya 1965, Miyatake 1966, Sasaji 1967, Hoàng 1982, Wang et al. 2010).

In this paper, a new species of *Nesolotis* from Laos was described, namely *Nesolotislaotica* **sp. nov.** A detailed description and illustration are provided, with the report of the new geographical record of *Nesolotis* in Laos.

## Materials and methods

The specimens used in the study were collected from Laos and deposited in the Department of Entomology, South China Agricultural University (SCAU).

Terminology follows Ślipiński (2007). The measurements were made using a micrometer attached to a SteREO Discovery V20 dissecting stereoscope and are defined as follows:

- **TL**—total length, from apical margin of clypeus to apex of elytra;
- **TW**—total width, across both elytra at widest part;
- **TH**—total height, through the highest point of elytra to metaventrite;
- **HW**—head width, including eyes;
- **PL**—pronotal length, from the middle of the anterior margin to the base of the pronotum;
- **PW**—pronotal width at widest part;
- **EL**—elytral length, along the suture, from the apex to the base including the scutellum;
- **EW**—elytral width, across both elytra at widest part;
- **ID**—interocular distance, the nearest distance between eyes.

The abdomen was detached and cleared in warm 10% sodium hydroxide (NaOH) solution for several minutes. Genitalia of both sexes were dissected, rinsed with distilled water, transferred to glycerol and examined on slides. Photographs were taken by using digital cameras (ZEISS Imager M2 and Axiocam 506 Color) attached to the dissecting microscope using the ZEN 2.3 software.

External morphological images were taken with a camera (Canon EOS 5D Mark IV) and processed by using Helicon Remote (v. 3.9.7W) and Helicon Focus 7.0.2 software.

## Taxon treatments

### Nesolotis laotica

Lv & Wang, sp. nov.

4C2EB91D-7B7E-5FF5-B3C8-F72652839D9B

AA8F1584-C734-4E2A-8B2A-C9A8BA77B8F1

#### Materials

- **Type status:** Holotype. **Occurrence:** catalogNumber: SCAU(E)10535; recordedBy: Wang XM, Telakang and Liang JB; sex: male; occurrenceID: 1A3807F2-9D5E-5775-96EC-0EE16C8FB742; **Taxon:** scientificName: *Nesolotislaotica*; class: Insecta; order: Coleoptera; family: Coccinellidae; scientificNameAuthorship: Lv & Wang; **Location:** country: Laos; municipality: Saleuy; verbatimElevation: 1340 m; decimalLatitude: 20.2316031; decimalLongitude: 104.0049098; **Identification:** identifiedBy: Lanlan Lv & Xingmin Wang; **Event:** year: 2007; month: 6; day: 9; **Record Level:** institutionID: South China Agricultural University; institutionCode: SCAU; basisOfRecord: Preserved Specimen
- **Type status:** Paratype. **Occurrence:** recordedBy: Wang XM, Telakang and Liang JB; sex: 1 male, 6 females; occurrenceID: 0D7A9781-331C-55E8-819F-0673755CB335; **Taxon:** class: Insecta; order: Coleoptera; family: Coccinellidae; **Location:** country: Laos; county: Na Khen; locality: Saleuy; verbatimElevation: 1340 m; decimalLatitude: 20.2316031; decimalLongitude: 104.0049098; **Identification:** identifiedBy: Lanlan Lv & Xingmin Wang; **Event:** year: 2007; month: 6; day: 9; **Record Level:** institutionID: South China Agricultural University; institutionCode: SCAU; basisOfRecord: Preserved Specimen
- **Type status:** Paratype. **Occurrence:** recordedBy: Telakang; sex: 1 male, 2 females; occurrenceID: 6C391DD6-8167-5D21-8FB2-30E8444BF94B; **Taxon:** class: Insecta; order: Coleoptera; family: Coccinellidae; **Location:** country: Laos; county: Paksong; decimalLatitude: 15.1771158; decimalLongitude: 106.2337374; **Identification:** identifiedBy: Lanlan Lv & Xingmin Wang; **Event:** year: 2005; month: 12; day: 1; **Record Level:** institutionID: South China Agricultural University; institutionCode: SCAU; basisOfRecord: Preserved Specimen
- **Type status:** Paratype. **Occurrence:** recordedBy: Lanlan Lv & Xingmin Wang; sex: 6 males, 1 female; occurrenceID: B4BD732F-ECD7-570B-9B14-F2C3480FE113; **Taxon:** class: Insecta; order: Coleoptera; family: Coccinellidae; **Location:** country: Laos; county: Paksong; decimalLatitude: 15.1771158; decimalLongitude: 106.2337374; **Identification:** identifiedBy: Lanlan Lv & Xingmin Wang; **Event:** year: 2006; month: 12; day: 2; **Record Level:** institutionID: South China Agricultural University; institutionCode: SCAU; basisOfRecord: Preserved Specimen
- **Type status:** Paratype. **Occurrence:** recordedBy: Telakang; sex: 1 female; occurrenceID: D18AAC6F-CC08-566D-8228-2DFB82E9623A; **Taxon:** class: Insecta; order: Coleoptera; family: Coccinellidae; **Location:** country: Laos; county: Paksong; decimalLatitude: 15.1771158; decimalLongitude: 106.2337374; **Identification:** identifiedBy: Lanlan Lv & Xingmin Wang; **Event:** year: 2005; month: 12; day: 5; **Record Level:** institutionID: South China Agricultural University; institutionCode: SCAU; basisOfRecord: Preserved Specimen
- **Type status:** Paratype. **Occurrence:** recordedBy: Telakang; sex: 1 male; occurrenceID: 6A4E0934-BC1A-59BB-A177-FFCC1323FF5F; **Taxon:** class: Insecta; order: Coleoptera; family: Coccinellidae; **Location:** country: Laos; county: Paksong; verbatimElevation: 1280 m; decimalLatitude: 15.1771158; decimalLongitude: 106.2337374; **Identification:** identifiedBy: Lanlan Lv & Xingmin Wang; **Event:** year: 2006; month: 6; day: 13; **Record Level:** institutionID: South China Agricultural University; institutionCode: SCAU; basisOfRecord: Preserved Specimen
- **Type status:** Paratype. **Occurrence:** recordedBy: Wang XM, Telakang & Liang JB; sex: 1 male; occurrenceID: C3D4E8D6-065B-5D2B-93DB-F19397148D14; **Taxon:** class: Insecta; order: Coleoptera; family: Coccinellidae; **Location:** country: Laos; county: Vientiane; municipality: Phonghong; verbatimElevation: 115m; decimalLatitude: 18.5055341; decimalLongitude: 102.4180054; **Identification:** identifiedBy: Lanlan Lv & Xingmin Wang; **Event:** year: 2007; month: 5; day: 16; **Record Level:** institutionID: South China Agricultural University; institutionCode: SCAU; basisOfRecord: Preserved Specimen

#### Description

TL: 1.54-1.6 mm, TW: 1.43-1.54 mm, TH: 0.95-1.1 mm, TL/TW: 1.04-1.08, EL/EW: 0.84-0.86, PL/PW: 0.40-0.47, HW/PW: 0.63-0.63, PW/EW: 0.71-0.72 EW/HW: 0.58-0.59.

**Figure 1.**
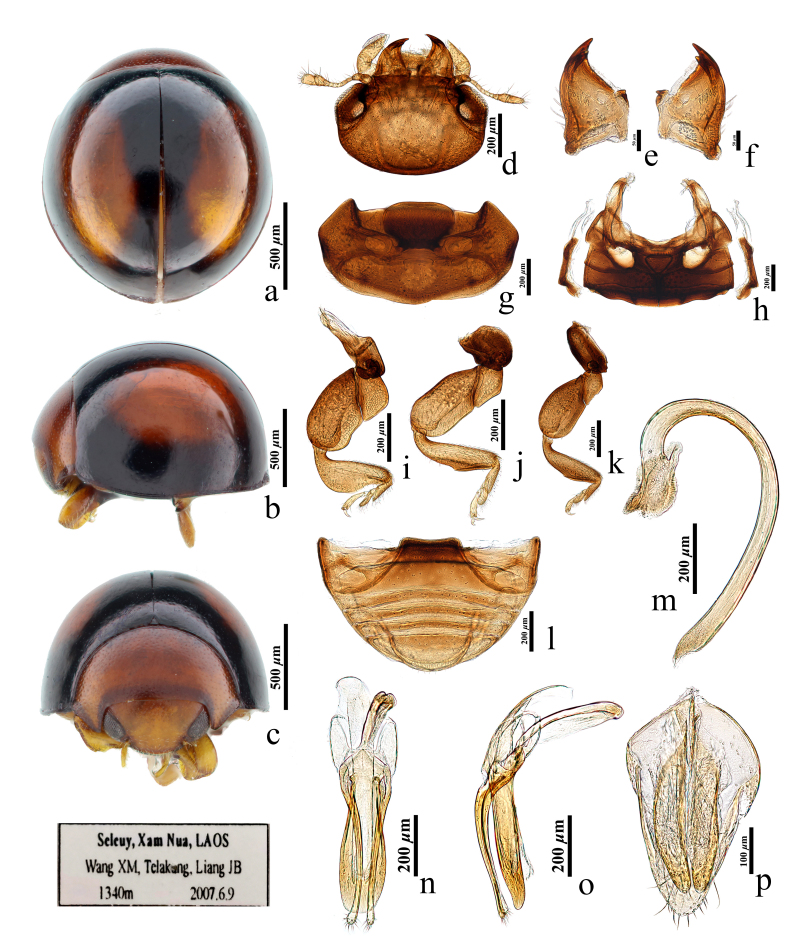
*Nesolotislaotica* Lv & Wang, sp. nov.: **a** dorsal habitus; **b** lateral habitus; **c** frontal habitus; **d** head, ventral; **e** left mandible; **f** right mandible; **g** prothorax; **h** mesoventrite and metaventrite; **i** front leg; **j** mid-leg; **k** hind leg; **l** male abdomen; **m** penis; **n** tegmen, ventral view; **o** tegmen, lateral; **p** coxite (genital plate).

Body brown. Head yellow, reddish-brown on the ventral side, paler than the thorax and abdomen (Fig. 1a-d, g and h). Prothorax reddish-brown, with anterior and lateral margins dark brown (Fig. 1g). Elytra dark brown, each elytron with an oval, yellow or yellowish-brown spot in the middle; the size of the spot varies amongst individuals, the larger spot nearly reaching the elytral margin (Fig. 1a and b). Legs generally yellow, coxa brown (Fig. 1i-k).

Body relatively small, nearly hemispherical, dorsum strongly convex above, shining without hair on the surface.

Head broad, subtriangular, about 0.45 times of the width of elytra (HW/EW = 0.7: 1.54), slightly retracted under pronotum, depressed downwards (Fig. 1d). Facet rough. The antenna expanded on basal and distal regions, with the first antennomere elongated, the second antennomere droplet-shaped and antennomeres 7-9 forming a fusiform club. Mandible robust, bifid at the apex, with one tiny and one larger pointed tooth (Fig. 1e and f).

Pronotum width about twice of the length, about 0.7 times of the width of the elytra (PW:EW=0.7:1.54), with densely fine punctures, which are similar to those on the head (Fig. 1g). Scutellum very small. Prosternum short, prosternal process very broad, distinctly broader than coxal diameter, trapezoidal and divergent anteriorly, with anterior corners round (Fig. 1g). Mesoventrite about as long as metaventrite, anterior margin strongly concave; junction of meso- and metaventrite slightly concave; metaventral postcoxal lines joined at the middle, recurved, present as W-shape; metaventrite with posterior margin slightly arcuate (Fig. 1g and h). Legs broad, femur and tibiae enlarged; tibiae of front leg strongly expanded on the outer side, slightly expanded on mid- and hind legs, with an abruptly tiny projection (Fig. 1o-q). Elytral surface polished, with extremely fine punctures, sparser than those on the head and vertex, mainly concentrated in the anterior 1/3 of the elytra (Fig. 1a and b). Hind wings well developed.

Abdominal postcoxal lines incomplete, without branching lines (Fig. 1a-b).

Male genitalia. Penis short and stout, curved at the basal 1/4, abruptly narrowing at tip, with membranous apex (Fig. 1m). Penis capsule swollen, with short inner and outer arms, almost equal in size (Fig. 1m). Penis guide stout and straight, slightly shorter than parameres, largely tongue-shaped, unparallel on both sides; in ventral view, slightly expanded in the middle, with obtuse tip; in lateral view, slightly narrowing towards the apex (Fig. 1n and o). Parameres slender and thin, slightly narrowing in the middle, slightly swollen distally, with a rounded tip, bearing short setae (Fig. 1n).

Female genitalia. Coxites symmetrically parallel, slenderly elongated, nearly crescent-shaped, about four times as long as wide and slightly narrowing at the tip (Fig. 1p). Spermatheca absent.

#### Diagnosis

This species can be distinguished from other species of *Nesolotis* by its dark brown elytron with an oval, yellow or yellowish-brown spot in the middle, the polished elytral surface with extremely fine punctures concentrated in the anterior 1/3 of elytra (Fig. 1a and b), the stout and short penis, the short inner and outer processes of penis capsule (Fig. 1m) and the tongue-shaped penis guide slightly shorter than parameres (Fig. 1n and o).

#### Etymology

The specific epithet “laotica” refers to the country, where the species has been discovered.

#### Distribution

Laos (Paksong, Na Khen, Vientiane).

##### Remarks

The genus *Nesolotis* was recorded from Laos for the first time. Amongst all the species of *Nesolotis*, *N.laotica* sp. nov. is similar to *Nesolotisobtusa* Wang & Ren, 2010 in having shining surface of the whole body, fine punctures mainly concentrated on anterior region of elytra, abdominal postcoxal lines without branching line and tongue-shaped penis guide, but it can be distinguished from the latter by the brown body with a pair of yellow to yellowish-brown, oval spots and the stout and short penis. In *N.* obtuse, the body is generally yellow and the penis guide is long and slender (Wang et al. 2014).

### Nesolotis

Miyatake, 1966

82417056-B29C-57A6-B6C1-63FA4DF7FC33

Nesolotis Miyatake, 1966 - Miyatake (1966): 47. Type species, original designation, *Nesolotisimpunctata* Miyatake, 1966.
Nesolotis : Sasaji, 1967 - Sasaji (1967): 16; Sasaji (1971): 75; Miyatake (1994): 264. Wang et al. (2010): 2; Wang et al. (2014): 21.
Paranesolotis Hoàng, 1982 - Hoàng (1982): 104. Type species, monotypy: *Paranesolotistamdaoensis* Hoàng, 1982, synonymised by Ślipiński (2004): 390.
Nesolotis as synonymy of *Sticholotis* Crotch: Ślipiński (2004): 390. Re-validated by Wang et al. (2010): 2.

#### Diagnosis

See Wang et al. (2010)

##### Remarks

*Nesolotis* shows high similarities with *Coelolotis* Miyatake, 1994 in external appearance, such as the strongly convex body, the densely fine punctures on head as well as on pronotum and elytra and the leg with front tibiae broadly expanded on outer margin (Miyatake 1994). It is noteworthy that *Nesolotis* was originally described with atrophied hind wing, a diagnostic feature that distinguished it from *Coelolotis*. However, based on the examination, the reduced hind wings are only present in certain island species, such as *Nesolotisimpunctata* and *Nesolotispunctifrons* Miyatake, 1966, while those species from mainland China exhibit normal-sized hind wings (Wang et al. 2010). Further investigation is necessary to discuss the systematic relationship between *Nesolotis* and *Coelolotis*.

#### Distribution

China, Japan, Vietnam, Laos.

## Supplementary Material

XML Treatment for Nesolotis laotica

XML Treatment for Nesolotis
